# “Can I Live”: Black American Adolescent Boys’ Reports of Police Abuse and the Role of Religiosity on Mental Health

**DOI:** 10.3390/ijerph17124330

**Published:** 2020-06-17

**Authors:** Ashley N. Jackson, Sheretta T. Butler-Barnes, Jewel D. Stafford, Helen Robinson, Phylicia C. Allen

**Affiliations:** 1 George Warren Brown School of Social Work, Washington University in St. Louis, St. Louis, MO 63130, USA; sbarnes22@wustl.edu (S.T.B.-B.); helenrobinson@wustl.edu (H.R.); allenpc@wustl.edu (P.C.A.); 2 College of Public Health and Social Justice, Saint Louis University, St. Louis, MO 63104, USA; jewel.stafford@slu.edu

**Keywords:** police abuse, public regard, religiosity, black male adolescents

## Abstract

State sanctioned violence aimed at Black individuals and communities is an issue that has pervaded American history and society since before the establishment of the United States. For Black males, anticipating and preparing for involuntary police contact, unfortunately, is an inevitable part of life. The purpose of this study is to examine the impact of reports of police abuse on mental health and perceived racial out-group perceptions and the protective role of religiosity among a nationally representative sample of Black American adolescent boys (M*_age_* = 14.98). Linear multiple regression was used to determine the interactive effects of subjective religiosity and reported police abuse on Black American adolescent boys. Higher reports of subjective religiosity were associated with lower depressive symptomatology. Reports of police abuse were associated with lower public regard beliefs (belief that society views Black Americans less favorably). Results highlight the impact experiencing police abuse has on Black adolescent boys and we conclude with implications, areas for future research and intervention points.

## 1. Introduction

The criminalization of and subsequent state sanctioned violence aimed at Black individuals and communities is a social issue that has permeated American society since the establishment of the United States [1]. From slave patrols aimed at maintaining white supremacy by terrorizing Blacks during slavery [2] to the killing of unarmed Michael Brown [3], minorities—and especially Black men—have been the target of police abuse at disproportionately higher levels than other racial and ethnic groups [4]. Empirical work has identified connections between historical legacies of the criminalization of Black males and its present proliferation [5,6], which continues to disproportionately effect Black males. In 2016, Terrence Cunningham—then president of the International Association of the Chiefs of Police (IACP)—recognized law enforcement’s role in perpetrating the abuse of minorities [7], though much is left to be achieved in terms of police reform and improving relations between law enforcement and racial and ethnic minorities. For Black males anticipating and preparing for involuntary police contact, unfortunately, is an inevitable part of life.

In 40 interviews with Black males aged 13 to 19, Brunson found that cumulative mistreatment was so common that respondents came to anticipate police abuse as a common occurrence in policing [8]. Subsequent studies also find that minority youth engage in avoidant behavior, evading certain places to reduce involuntary encounters with the police [9,10,11].

Despite these circumstances, mechanisms may be activated to combat the adverse effects on psychological well-being as a result of negative police contact. Religiosity, in particular, may play a buffering role against the adverse effects and out-group perceptions of those who have experienced police abuse, particularly among Black youth during some of the most formative years in adolescent development. The role of religion in addressing mental health among minority adolescents, however, is complex. When Rose and colleagues explored the role of religion in connection to the mental health of Caribbean Black adolescents they found that involvement in organized religious activities was positively related to coping and self-esteem but negatively related to depression and attending religious service was not related to any mental health domains [12]. Qualitative work exploring the role of religion among African American adolescents also found that religion and spirituality played a salient role in addressing depression [13]. Earlier work also found that adolescent girls were more likely to value religion than boys [14], highlighting the need for further research exploring the complex ways religion becomes a salient part of the developmental processes of adolescent youth particularly in connection to police abuse. Additionally, less studied are the ways religiosity may alleviate the negative consequences of police abuse among adolescent minority youth.

### 1.1. Police Abuse of African Americans and Caribbean Blacks

A meta-analysis of the factors associated with officers’ decision to use force finds that the characteristics of the individual targeted by police abuse plays an important role in the likelihood of police use of force. Specifically, minorities, males, and/or individuals from economic disadvantage were most likely to experience police use of force [15]. Recent work also highlights that racially biased policing, “the hoodie effect”, and stand your ground laws may also be potential explanations for police and civilian abuse carried out against minorities [16].

Recent empirical work on police abuse found that, when compared to Whites, Blacks are more likely to be fatally shot by law enforcement [17]. While some work has found that women with a history of intimate partner violence and sexual violence [18] and Black women with income levels over $50,000 [19] experience high levels of police abuse, at the national level, Black males are still more likely to experience police abuse [4,19]. Using the African American and Caribbean black adult sample of the National Survey of American Life (NSAL), Oh and colleagues used a questionnaire adapted from the Lifetime Discrimination subscale of the Detroit Area Study Discrimination Questionnaire [20], and among the nine primary discriminatory events experienced, police abuse was the most commonly reported form of discrimination [21].

Empirical pursuits exploring police violence have suffered from challenges around its operationalization both historically and presently. For example, during the 1800s, the U.S. lacked any sort of nationally mandated tracking of lynching victims. As a result, the sociological and journalistic work of Ida B. Wells uncovered the occurrence and prevalence of lynching, as made evident in her development of the *Red Record*, which systematically tracked accounts of lynching reported in the *Chicago Tribune* [22]. Presently, no systematic government infrastructure exists that tracks the number of individuals experiencing police violence [23]. Nonetheless, crowd sourced data sources of police violence have emerged in recent years to fill these gaps [24,25].

### 1.2. Racial Identity among African American and Caribbean Black Youth

Racial identity is one’s attitude, beliefs and self-concept regarding the meaning and significance they ascribe to their racial group membership [26]. Furthermore, because racial identity arises from a group’s common history and shared values [27], racial identity for African Americans has been and continues to be coercively shaped by the discriminatory and oppressive experiences of racism [28]. Scholars have argued that this is particularly salient for African American youth [29]. A major contribution to the research that examines how African American youth perceive their racial group membership and how these perceptions shape experiences is the seminal work of Sellers and colleagues in their development of the Multidimensional Inventory of Black Identity (MIBI) scale [30] and its adaptation for African American adolescents [31]. An empirical tool used specifically for Black/African American populations, the MIBI scale provides a means to capture and assess racial identity across three cross-situational dimensions—centrality, regard, and ideology. Racial centrality is the extent to which one’s self concept is informed by their race and regard. Private regard refers to the assessment, either positive or negative, an individual makes about being an African American as well as differential feelings towards other African Americans [32]. Additionally, public regard is the extent to which one determines how other racial groups view their racial group [32].

Research shows that endorsement of a healthy racial identity is associated with more positive mental health outcomes [33]. Research also finds that increased racial identity is associated with decreased depressive symptomatology underscoring the important role of racial identity in addressing psychological distress among African American adolescents [33,34]. Findings that examined the relationship between racial identity beliefs and mental health outcomes among older African American and Caribbean blacks have found that endorsing higher levels of feelings of closeness to Blacks was associated with lower depressive symptomatology for Caribbean black older adults [35]. Additionally, lower evaluations of Black groups (endorsing negative stereotypes about Blacks) were associated with higher depressive symptomatology [35].

Thus, we extend the racial identity literature by examining how reports of police abuse among Black American boys impact public regard beliefs (e.g., perceptions of positive and negative views out-group members hold about one’s racial ethnic group). For instance, Sellers and colleagues [33] found that experiencing higher levels of racial discrimination and endorsing higher public regard beliefs was associated with higher depressive symptomatology and psychological distress among African American adolescents [33]. Additionally, Black populations are not monolithic, as they experience these outcomes in unique ways. Unfortunately, the theoretical, empirical and methodological approaches examining how racial variations and cultural differences influence health behaviors within black populations are limited [36]. There is variability in black immigration trends, for example, that collectively contribute to the heterogeneity of U.S. black population groups. While the recent increase in the Black population in the U.S. is due in part to African immigration, Caribbean countries—namely Jamaica and Haiti—have fueled most of the upticks in the number of Black immigrants to the U.S. [37]. Migration trends and acculturation to the U.S. culture may present different implications for Caribbean black adolescents in particular.

### 1.3. Mental Health of African American and Caribbean Blacks

The compounding effect of police abuse only further exacerbates health and mental health challenges. A large body of work exploring the adverse effects of aggressive policing highlights the ways these encounters are associated with several mental health challenges [38,39]. Research also shows that living in a heavily policed community is associated with psychological distress not only at the micro level [40], but also at the macro (e.g., community level) [41]. While reports of mental disorders are typically lower among Blacks compared to Whites [42], recent cohort studies show that reports of anxiety disorders have increased over time for Black individuals [43]. Compared to Whites, while Blacks have a lower lifetime risk of mood and anxiety disorders when factoring in economic disadvantage, Blacks are more likely to experience mood and anxiety disorders [42]. Subsequent empirical work further supports this by unpacking the within-group differences among Blacks, finding that Caribbean Black men were more at risk than African American men for developing psychiatric disorders [21,44,45]. Immigration status plays a critical role among first-generation Caribbean Blacks showing lower rates of psychiatric disorders compared to second- and third-generation Caribbean Blacks [45]. In a study of 570 African American men and women, African American men reported higher accounts of discrimination compared to African American women with findings also showing that perceived discrimination was associated with depression and anxiety [44].

Another study exploring associations between police abuse and psychological distress among the NSAL adult sample find that African Americans were more likely to report hallucinations compared to Caribbean Black adults [21]. Additionally, for both African American and Caribbean Black adults, police abuse was the most commonly reported form of discrimination—as previously mentioned—but police abuse, specifically, was the only factor that was significantly associated with an increased risk for lifetime psychotic experiences when adjusting for demographic factors [21]. While findings were not broken down by gender, findings also showed that in addition to being denied upward employment mobility and being discouraged from pursuing educational advancement opportunities, police victimization was also associated with lifetime hallucinations [21].

There is a growing body of work exploring mental health outcomes for Black youth using the National Survey of American Life (NSAL-A) adolescent sample exploring issues around perceived discrimination and psychological distress [46,47], with one study finding differences across gender [48]. When exploring perceived discrimination among African American and Caribbean Black youth, lower reports of depressive symptomatology was found among Caribbean Black youth with immigrant parents compared to African American youth [47]. However, lower perceptions of discrimination were also found among Caribbean Black youth with foreign born parents compared to Caribbean Black youth born to U.S.-born parents [47]. Additionally, while higher levels of perceived discrimination was associated with mental health in general, stronger associations were found between perceived discrimination and mental health constructs for Caribbean Black youth with foreign born parents compared to African American youth [47]. Other work analyzing the NSAL youth data, found that a large majority of Caribbean Black youth experienced discrimination which was associated with depression and anxiety, and higher levels of reported discrimination was associated with an increased rate of anxiety disorders [46]. Gender differences also emerged in another study that analyzed the NSAL youth data, with findings showing that African American and Caribbean Black youth perceived at least one incident of discrimination in the previous year, with males perceiving more incidents of discrimination than females [48]. Positive associations were also found between perceptions of discrimination and depressive symptoms with Caribbean Blacks being most at risk for perceiving higher accounts of discrimination [48].

### 1.4. The Important Role of Religion

Compared to other adolescents, African American adolescents are the most religious group in the United States, as indicated by prayer frequency and importance of religion [14]. Butler-Barnes, Martin, and Boyd [49] found that African American adolescents who reported a higher communication with God (e.g., I am receptive or open to God) were more likely to report a healthier psychological well-being. Associations have also been found between collaborative religious coping styles (e.g., seeking God for guidance) and higher depression, hopelessness, and suicidal attempts among African American adolescents [50]. Pearce, Little, and Perez [51] examined religiosity and the impact on depressive symptomatology among a diverse sample of adolescents. The findings revealed that positive interpersonal experiences (e.g., perceived support provided by the congregation) were associated with lower levels of depressive symptomatology. Le, Tov, and Taylor [52] examined the relationship between religiosity and depression among a nationally representative sample of adolescents within the U.S. and found that religiosity was associated with lower levels of depression and higher reports of self-esteem for African American adolescents. Results from focus groups with African American adolescents also revealed religious and spiritual coping themes indicating the important role of religion in coping with depression [13]. Yet, despite the important role of religion in the lives of adolescents and most importantly Black adolescents, a systematic review found that few studies have examined the role of religion in the lives of African American adolescents (see Cotton et al. [53]). Moreover, in addition to the large body of research on the important role of religion in the lives of Black adolescents, majority of these findings highlight the important role of religion for African American youth, assuming that Black adolescents are a monolithic group. Studies examining the important role of religiosity among Caribbean black adolescents have found similar findings on the importance of religion. Rose, Finigan-Carr, and Joe [12] found that 62% of Caribbean black adolescents attended church services and approximately 50% participated in a youth-based religious activity. Rose et al. [12] also found that religious emotional support and participation in church-related activities was associated with coping. Additionally, for Caribbean black adolescents, under conditions of experiencing higher racial discrimination, higher church attendance and higher reports of prayer was associated with lower depressive symptomatology [54]. Hope et al. [55] examined religious social support and psychiatric disorders among Black adolescents and found that religious support was associated with being less likely to meet the criteria for a psychiatric disorder among a sample of Black American youth. With regard to gender, Black girls have been reported to be more religious than Black boys [14], and studies have examined the role of religiosity among Black girls and psychological well-being [56,57]. In the current study, we build on the previous literatures by examining the protective role of religion in the lives of Black American adolescent boys.

### 1.5. Aims of This Study

Guiding this study is the Integrative Model for the Study of Developmental Competencies of Minority Children, which argues that there are sociocultural processes influencing the developmental processes of adolescents of color [58]. The model’s underlying assumptions are that social categorizations like class, race or ethnicity come with differential forms of “spatial, physical, social and psychological environments”, and that we develop a hierarchical system of attitudes and beliefs about the self and others who are either higher or lower in social class system (p. 1897) [58]. Our study uses this framework to explore how experiencing police abuse impacts public regard beliefs (e.g., how others view one’s racial ethnic group membership) and psychological well-being of African American and Caribbean Black adolescent boys and how this differs across socioeconomic status and age. We also seek to explore how experiencing police abuse interplays with religion given its importance in the sociocultural and developmental processes of Black adolescents. Moreover, this framework serves as a guide to position Black American adolescent boys’ everyday lived experiences in communities.

Due to the vast reports of racial profiling [59] and police abuse [60,61] and overall negative encounters with law enforcement [62], we seek to understand how communities can serve as promotive (positive) or inhibitive (negative) settings that shape Black American adolescent boys developmental competencies, specifically mental health and racial identity beliefs. In particular, we seek to understand how reports of police abuse impact depressive symptomatology, perceived stress and how others in society view their racial ethnic group membership (e.g., public regard beliefs). Based on the environments (e.g., neighborhoods) that minority children interact with, they, along with their families, make decisions on how to operate within these settings. For example, families prepare their children in anticipation for inhibitive environments (e.g., those that exacerbate racial discriminatory experiences) to protect themselves, giving them the efficacy needed to combat these experiences [63]. This Integrative Model for the Study of Developmental Competencies of Minority Children illuminates how these sociocultural processes manifest. Thus, we hypothesize that among adolescent Black American boys, experiencing police abuse will have adverse effects on psychological well-being and public regard beliefs. We also posit that religion will buffer against the negative impact of police abuse. To test these hypotheses we aim to examine the following: (1) Does reported police abuse impact depressive symptomatology, perceived stress, and public regard beliefs? (2) Does endorsement of higher subjective religiosity predict depressive symptomatology and perceived stress?; and (3) Does subjective religiosity serve as buffer in reducing the negative impact of reported police abuse on mental health and public regard beliefs?

## 2. Methods

Data for this analysis are from the National Survey of American Life—Adolescent Supplement (NSAL-A), a nationally representative household survey estimating prevalence, frequency, and comorbidity of DSM-IV disorders among 1170 African American and Caribbean adolescents ages 13–17 connected to adult households from the National Survey of American Life (NSAL) adult sample. The NSAL adult sample was part of the National Institute of Mental Health (NIMH) Collaborative Psychiatric Epidemiology Survey (CPES) which consists of the NSAL, the National Comorbidity Survey Replication (NCS-R), and the National Latino and Asian American Study (NLAAS) [64]. Additionally, the NSAL-A data tracks risk and protective factors for the onset of DSM-IV disorders and service use and tracks several measures including health, social conditions, stressors, racial identity, neighborhood conditions, activities and school, media and social and psychological protective and risk factors [64]. The data were weighted to adjust for non-independence and non-response rates in selection probabilities across households and individuals. The weighted data were post-stratified to approximate the national population distributions for gender (males and females) and age (13, 14, 15, 16, and 17) among African American and Caribbean Black youth.

Informed consent and assent was obtained from both the adolescent’s legal guardian and the adolescent. Most of the adolescent interviews were conducted face-to-face using a computer-assisted instrument in their homes, while other interviews were either conducted on the phone or partially (approximately 18% face-to-face or phone interview). Overall, interviews lasted 1 h with an overall response rate of 80.6% (80.4% for African Americans and 83.5% for Caribbean Blacks).

### 2.1. Measures

*Demographic*. Annual household income (1 = 0 to $10,000, 2 = $10,001 to $25,000, 3 = $25,001 to $50,000, 4 = $50,001 to $75,000, 5 = $75,001 to $100,000, 6 = over $100,000; reported by parent), race (0 = African American & 1 = Caribbean Black), and adolescent ages were included.

*Police Abuse.* Police abuse was measured using a dichotomous variable (1 = Yes and 0 = No) in response the question *“have you ever been abused by the police?”*.

Subjective Religiosity. Subjective religiosity was measured with two items: “How important is religion in your life?” and “How important is prayer when you deal with stressful situations?” Item responses ranged from 1 = very important to 4 = not important at all. Responses were reverse scored and averaged so that higher scores on the scale reflected higher religiosity beliefs. Internal consistency for the scale is 0.71.

*Mental Health.* Depressive symptomatology was measured using an abbreviated 12-item version of the Center for Epidemiologic Studies Depression (CESD) Scale [65,66]. The CES-D 12 item version includes major domains of depressive symptomatology including negative affect, positive affect, somatic symptoms, and interpersonal challenges [65,66]. Item responses ranged from 0 = rarely or never to 3 = all the time. Higher scores on the scale reflected levels of depressive symptomatology. The internal consistency for this scale is 0.64. Cohen’s Perceived Stress scale [67] was used to assess everyday stressors. The scale is comprised of 14 items (e.g., “felt nervous and stressed out?”, “gotten angry because of things that happened that were outside of your control?”). Item responses ranged from 1 = never to 5 = very often. Higher scores on the scale reflected higher levels of perceived stress. The internal consistency for this scale is 0.74.

*Racial Identity Beliefs*. Racial identity beliefs are comprised of seven subscales of the Multidimensional Inventory of Black Identity scale containing three domains of African American racial identity including ideology, centrality, public and private regard [30]. For the current study, we used the public regard subscale, which was comprised of four items (e.g., “In general, other racial groups view Blacks as competent people”, and “In general, other racial groups view Blacks in a positive manner”). Item responses ranged from 1 = strongly disagree to 4 = strongly agree. Scores were averaged, and higher scores on the scale reflected higher levels of positive public regard beliefs (viewing Black more favorably). The internal consistency for this scale is 0.77.

### 2.2. Analysis

MPLUS 7.3 was used to conduct the analyses because of the complex design-based estimates of variance. The weighted data were used. The NSAL-A sample is a stratified and clustered sampling design. Full-Information Maximum Likelihood (FIML) estimation was used to account for missing data. Approximately 71% of the sample was African American and 29% were Caribbean Black adolescent boys. We used linear multiple regression analyses for multivariable data. In the model, reports of police abuse (1 = yes and 0 = no), subjective religiosity, and the subjective religiosity x police abuse interaction were the main predictors in the model. Depressive symptomatology, perceived stress, and public regard were the outcomes and adolescent age, household income, and race (0 = African American and 1 = Caribbean Black) were covariates in the model.

## 3. Results

### 3.1. Sample Characteristics and Correlations of Black American Adolescent Boys

Sample descriptive statistics and correlations are presented in Table 1 and Table 2, respectively. The mean age of sample participants was 14.98 (SE = 0.06), range = 13–17). The majority of participants were African American (71%) and Caribbean Black boys (29%). Overall, Black American adolescent boys reported lower levels of depressive symptomatology, moderate levels of positive public regard beliefs, moderate levels of perceived stress, and lower levels on average on subjective religiosity (Table 1). Adolescent age was statistically negatively associated with public regard beliefs (*r* = −0.15, *p* < 0.10), and family income was statistically negatively associated with perceived stress (*r* = −0.11, *p* < 0.10) (Table 2). Additionally, 26% of the youth in the sample had been abused by the police.

### 3.2. Regression Results for Black Adolescent Boys

Linear regression analyses were performed to examine the demographics (i.e., age, ethnicity, and household income), independent variables (reported police abuse and subjective religiosity), and the interaction effect of subjective religiosity x reported police abuse on the mental health and public regard beliefs of Black American boys. All of the variables were simultaneously entered into the model to examine the impact on three outcome variables: depression, public regard beliefs, and perceived stress. Table 3 presents the order in which the variables were entered and regressed on mental health outcomes and public regard beliefs. Separate columns are presented for depressive symptomatology, public regard beliefs, and perceived stress.

In model 1, controlling for the covariates (i.e., age, ethnicity, and household income), subjective religiosity was associated with lower reports of depressive symptomatology (*B* = −0.010; *SE* = 0.002, *p* = 0.001). In model 2, after controlling for the covariates, household income (*B* = −0.048; *SE* = 0.025, *p* = 0.049) and reported police abuse (*B* = −0.305; *SE* = 0.135, *p* = 0.024) was associated with public regard beliefs. More specifically, Black American boys who reported being abused by the police felt that society did not view them favorably in comparison to those who did not report being abused by the police. In model 3, controlling for covariates, reported household income (*B* = −0.856; *SE* = 0.270, *p* = 0.002) was associated with perceived stress. Black American boys reporting higher household income was associated with lower levels of perceived stress. Finally, the subjective religiosity x police abuse interaction for models 1 to 3 was not statistically significant for depressive symptomatology, public regard, or perceived stress.

## 4. Discussion

Our study sought to explore how experiencing police abuse impacts psychological well-being (e.g., depressive symptomatology and perceived stress) and public regard beliefs among Black American adolescent boys and the role of subjective religiosity in buffering against the negative effects of police abuse. We posited that among adolescent Black American boys, experiencing police abuse would negatively impact psychological well-being and public regard beliefs and that subjective religiosity would buffer against the negative effects of police abuse. Our findings are provided in the following sections, and overall show partial support of our hypothesis.

### 4.1. Depressive Symptomatology

Our hypothesis that experiencing police abuse impacts depressive symptomatology was not supported; however, our hypothesis that subjective religiosity would be associated with lower depressive symptomatology was supported. More specifically, findings did reveal that, overall, subjective religiosity was associated with lower levels of depressive symptomatology. This particular finding highlights how the specific construct of religion may reduce negative mental health outcomes and aligns with prior work that finds associations between religiosity and healthy psychological well-being [13,49].

Our findings also corroborate previous work on the important role of subjective religiosity in the lives of Black Americans [68,69,70]. This particular finding highlights how the specific construct of religion may reduce negative mental health outcomes and aligns with prior work that finds associations between religiosity and healthy psychological well-being [13,49]. For instance, Taylor, Mattis, and Chatters [70] found that African American adults reported higher levels of subjective religiosity in comparison to White Americans. Thus, our work builds on previous research by documenting the important role of subjective religiosity among Black American adolescent boys.

### 4.2. Public Regard

In support of our hypothesis that police abuse would impact the public regard beliefs of Black American adolescent boys, the results showed lower levels of public regard (e.g., perception that society views one’s racial group less favorably) among Black American adolescent boys who were abused by the police. These particular findings highlight the complexity of the lived experiences of minority youth and contribute to the variations found in empirical work regarding the within-group differences among minority groups relative to police contact. For example, while some studies have found that foreign-born participants tend to hold more favorable views of the police when compared to U.S.-born participants [71,72], other studies have found that foreign-born individuals held less favorable perceptions of the police both at the individual [73] and neighborhood level in communities with greater numbers of foreign-born residents [74]. In our study, the findings revealed no statistically significant differences with respect to police abuse and public regard beliefs when controlling for ethnicity. These findings highlight the possibility that experiencing police abuse particularly during one of the most formative developmental periods in a Black American adolescent boy’s life may hold more salience then one’s familial place of origin.

Lastly, we found that higher household income was associated with Black American boys perceiving that others in society viewed their racial group less favorably. We speculate that because of Black American boys’ racialized status within the U.S., higher socioeconomic status does not protect them from negative racial encounters and experiences. For instance, recent research has found that higher SES does not protect Black men against depression [75] and that upward social mobility among Black Americans is associated with higher physical health problems [76]. Lastly, Wikrama, O’Neal, and Lee [77] found among a nationally representative sample of adolescents that striving for higher SES attainment was predictive of higher stress levels. Overall, additional research is warranted to further understand the intersection of socioeconomic status and identity among Black American adolescents.

### 4.3. Perceived Stress

Our hypothesis that experiencing police abuse and subjective religiosity would impact levels of perceived stress was not supported. However, we found that socioeconomic status played a key role as Black American adolescent boys from households with higher incomes reported lower levels of perceived stress. This finding contributes to earlier work indicating that low-income Black youth reported higher levels of psychological distress [78]. Unemployment has also been found to be associated with depression, particularly among African American men [79]. This finding points to the importance of socioeconomic status on stress across the life course particularly as Black American adolescent boys navigate through adolescence, emerging adulthood, and later to adulthood.

### 4.4. Limitations

A limitation of the study is that the National Survey of American Life Adolescent Supplement was collected from 2001 to 2004. While it is possible that the experiences of youth abused by the police and associated effects of religiosity and mental health may vary from the experiences of minority youth presently, many of these challenges may still remain. Another limitation is the low occurrence of police abuse among the adolescent youth in the sample (26%). We suspect that this smaller sample size might be due to the phrasing of the question (e.g., have you ever been abused by the police?), which highlights the challenges around how police abuse is operationalized empirically. Measuring police abuse faces similar challenges to those faced by intimate partner violence (IPV) research with respect to what should be included in the definition of IPV, from physical to emotional and psychological abuse [80]. The structure of the NSAL-A survey question regarding police abuse is broad (e.g., 1 = Yes and 0 = No, in response the question “have you ever been abused by the police”), possibly leading to a misunderstanding that the term ‘police abuse’ may include other forms of abuse in addition to physical, such as emotional and psychological abuse and neglect. Recent work using the novel Police Practice Inventory (PPI) measured victimization with the police across a variety of domains of abuse informed by the World Health Organization (WHO) and included physical victimization (with and without a weapon), psychological victimization, sexual victimization, and neglect [81]. It is possible that youth in the NSAL-A sample may have experienced other forms of police abuse such as the types laid out in tools like the PPI which may explain the low sample size.

Additionally, the cross-sectional design of the NSAL-A limits an aspect of our study’s aim to explore the impact of police abuse on the developmental processes of adolescents over time. This design also makes it difficult to discern the trajectory of mental health over time with regards the psychological well-being of Black American adolescents who have experienced police abuse. Place matters, especially with respect to the effects and prevalence of negative police encounters. Neighborhood indicators of social cohesion, social disorder, and level of quality of community-police relations in a neighborhood may have shed important light on the psychological well-being outcomes particularly among minority adolescents and their interactions with local law enforcement.

## 5. Implications

Several implications arose from this study. First, research on trauma and brain development continues to emerge, pointing to the specific ways trauma can affect brain development, particularly when trauma occurs early in life [82]. Future research is needed that explores the adverse effects of experiencing police abuse, both directly and vicariously through a family member or through exposure to police abuse at the community level. This future research is crucial given the increased accessibility of information regarding violent police encounters online [83] and the potential role social media plays in raising awareness to police abuse impacting minorities [84,85]. Our findings regarding the adverse effects of police abuse on public regard among youth also highlights key constructs of Coll’s Integrative Model. Specifically, this finding underscores the traumatic effects of police abuse experienced during adolescence and its long-term effects, given its occurrence during some of the most formative years in youth development.

Our findings also highlight how punitive responses by institutions aimed at minorities do not operate in a vacuum. Differential treatment of youth is widely known in educational institutions, perpetrated through School Resource Officers (SROs) [86] and zero tolerance policies [87], laying the groundwork for a school to prison pipeline [88]. Punitive treatment by institutions is an experience that spans the life course, beginning as early as preschool. Black male youth in the U.S. receive punitive actions at higher levels than other racial groups in myriad ways from school discipline [89] to contact with the criminal justice system [90]. Black male youth are also suspended from pre-school through grades K through 12 at higher levels than their white male counterparts [89]. More research is needed exploring how the penal responses of schools might serve as a pathways to exposure to police abuse later in adolescence.

Additionally, a meta-analysis examining the role of racial identity and academic achievement found a small but significant effect size linking racial identity with academic achievement [91]. Recent research conducted on over 200,000 adolescents in New York City found that aggressive policing reduced test scores for African American boys [92]. Thus, more research is needed investigating the ways police abuse, specifically, is associated with academic achievement and subsequent socioeconomic advancement. While our sample highlighted the experiences of youth impacted by police abuse during the early oughts, these experiences also hold value. Connections can be drawn between the youth in this study’s sample to that of the experiences of minority youth in present time, given the shifting political landscape namely with the rise of the Black Lives Matter Movement and the development of the President’s Task Force on 21st Century Policing developed under the Obama administration [93].

Additionally, more longitudinal research is needed to understand how police interactions impact Black American boys’ developmental competencies and self-perceptions over time. Black American adolescent boys who reported police abuse in the current study perceived that society held more negative views about their racial group membership which may impact other areas of development such as perceived self-worth. Future research is needed that deploys mixed methodologies to understand the lived experiences of Black American boys relative to interactive effects of law enforcement, religiosity, and the role of additional support mechanisms (e.g., peer support, racial socialization, and parent support). More specifically, qualitative research is warranted to better understand the affective meaning of these negative encounters with the police and its impact on Black American boys’ sense of self.

Halgunseth and colleagues found variation in the significance of religious beliefs and practices of African American parents on their children, with mothers’ religious beliefs being linked to the beliefs of both their sons and daughters and found that fathers’ religious beliefs were only linked to sons’ religious beliefs [94]. However, when taking into account a parent’s religious practices, mothers with moderate religious practices was more strongly linked to youth religious practices and fathers’ religious practices was stronger for their daughters [94]. This would also aid in unpacking the nuanced meaning of the importance of religion or spirituality in the lives of Black American adolescent boys.

The null finding related to police abuse, perceived stress, and depressive symptomatology, compared to the statistically significant association found between public regard and reported police abuse could be due to barriers Black Americans face with respect to mental health. Specifically, Thompson and colleagues found that stigma, costs, cultural misunderstanding, and trust were major barriers among African Americans seeking mental health services [95]. Additionally, stereotypes and portrayals of Black individuals influence how minorities are perceived [96]. These barriers to accessing quality mental health services in combination with the pressures that come with being a member of a highly stereotyped group, might explain why Black adolescents place more value on public regard beliefs rather than perceived stress and depressive symptomatology. These barriers to mental health, coupled with other empirical work showing prayer was a critical coping mechanism among Black women, highlights the essential role religion plays among Black individuals. Our findings around the role of religiosity in addressing depressive symptomatology highlight the positive impact religiosity plays over the life course. However, it also calls for future research examining the role of religiosity—both non-organizational religiosity and spirituality—among Black American adolescent boys to further understand how religion and spirituality shapes other developmental competencies (e.g., self-esteem, self-worth, and academic achievement) in connection with police abuse.

## 6. Conclusions

Police abuse involving minorities, particularly Black men, has been a haunting feature of U.S. history. These issues have once again caught national attention most recently with death of George Floyd, Breanna Taylor and countless others prompting global protests, recognitions by police organizations of the historical mistreatment by the police of minorities [7], and national commitments to improving community–police relations [93]. Nonetheless, the incessant classification of Black bodies as criminal and dangerous [61] makes it understandable why Black youth engage in several behaviors like avoidance to reduce chance encounters with the police [9,10,11]. The fields of social work, public health, criminal justice, and other youth serving entities lack sufficient interventions or strengths-based approaches to more effectively support Black males experiencing police abuse and its associated adverse effects. While Black males are often targets of aggressive policing, they are also the most likely to be the victims of a crime and seek support through informal social supports, rarely relying on formal institutions for assistance (e.g., police, therapeutic support, etc.) [95,97,98]. Our findings, nonetheless, provide a promising area to explore for interventions or programming to address mental health challenges by tapping into minority youth perspectives on religious engagement to explore what specific aspects of religion and spirituality hold more salience. The novel intervention—Racial Encounter Coping Appraisal and Socialization Theory (RECAST)—implemented by Anderson and Stevenson offers a promising step toward healing among minorities who have experienced traumatic events like negative police encounters. [99]. Ultimately, our findings not only highlight the nuanced effects police abuse has on public regard but also lay the groundwork for potential intervention points, namely the use of religiosity and approaches to enhancing racial identity, as ways to improve overall mental health for minority youth exposed to police abuse.

## Figures and Tables

**Table 1:** Descriptive statistics for Black American adolescent boys.

Variable	M	SE
1. Age	14.98	0.06
2. Income	2.70	0.10
3. Religiosity	1.64	0.16
4. Depression	8.96	0.29
5. Public Regard	2.98	0.04
6. Perceived Stress	34.36	0.38

Household income ranged from 1 = 1 to $10,000 to 6 = over $100,000 per year. Average household income was 25,001 to 50,000.

**Table 2:** Correlations for Black American adolescent boys.

Variable	Correlations				
1. Age	1	2	3	4	5
2. Income	0.064				
3. Religiosity	0.058	0.027			
4. Depression	−0.027	−0.060	−0.068		
5. Public Regard	−0.150 **	−0.061	−0.001	0.001	
6. Perceived Stress	−0.012	−0.119 **	−0.025	0.596 **	−0.017

** *p* < 0.10. Household income ranged from 1 = 1 to $10,000 to 6 = over $100,000 per year. Average household income was 25,001 to 50,000.

**Table 3:** Linear regression analysis for variables predicting depressive symptomatology, public regard, and perceived stress among Black American adolescent boys.

Variable	Model 1			Model 2			Model 3		
	Depressive Symptomatology			Public Regard			Perceived Stress		
	*B*	*SE B*	*p*-Value	*B*	*SE B*	*p*-Value	*B*	*SE B*	*p*-Value
Age	−0.035	0.185	0.852	−0.035	0.025	0.162	0.056	0.268	0.834
Ethnicity	−0.087	1.41	0.538	−0.076	0.237	0.749	2.33	1.61	0.148
Income	−0.035	0.200	0.077	−0.048	0.025	0.049 *	−0.856	0.270	0.002 **
Police Abuse	0.507	1.50	0.735	−0.305	0.135	0.024 *	1.64	2.48	0.508
Religiosity	−0.010	0.002	0.001 ***	0.001	0.001	0.454	−0.002	0.003	0.405
Religiosity x Police Abuse	0.002	0.791	0.998	0.055	0.073	0.446	−0.561	1.12	0.616

Ethnicity 1 = Caribbean Black and 0 = African American, Police Abuse 1 = Yes and 0 = No. * *p* < 0.05, ** *p* < 0.010, *** *p* < 0.001. Model 1 = Depressive Symptomatology, Model 2 = Public Regard, and Model 3 = Perceived Stress.
